# 45,X[2]/46,X,der(Y).ish Psu idic(Y)(q11.2)[38] mosaic karyotype in mixed gonadal dysgenesis: a case report and literature review

**DOI:** 10.3389/fped.2024.1460174

**Published:** 2024-10-16

**Authors:** Qiang Zhang, Xiaoxiao Chen, Yanyan Cao, Yun Zhou, Yingye Liu, Lijun Liu, Lei Liu, Xiaowei Cui

**Affiliations:** ^1^ Department of Endocrinology, Genetics and Metabolism, Children’s Hospital of Hebei Province, Shijiazhuang, Hebei, China; ^2^ Pediatric Clinical Research Centre of Hebei Province, Children’s Hospital of Hebei Province, Shijiazhuang, Hebei, China; ^3^ Institute of Pediatric Research, Children’s Hospital of Hebei Province, Shijiazhuang, Hebei, China; ^4^ Department of Urology, Children’s Hospital of Hebei Province, Shijiazhuang, Hebei, China; ^5^ Department of Ultrasonography, Children’s Hospital of Hebei Province, Shijiazhuang, Hebei, China

**Keywords:** isodicentric Y chromosome, mosaicism, Yq11.2 breaks, mixed gonadal dysgenesis, karyotyping, fluorescence *in situ* hybridization

## Abstract

Mixed gonadal dysgenesis is caused by a variety of chromosome abnormalities, most commonly Y chromosome mosaicism. An 8-year-old boy presented with short stature for possible treatment with recombinant growth hormone. He had a history of mixed gonadal dysgenesis (hypospadias, bilateral cryptorchidism, processus vaginalis, and dysplastic immature uterus) and a series of corrective surgeries. At 14 months of age, chromosomal karyotyping revealed 46,X,+mar. Upon presentation, lab testing was consistent with the male phenotype at prepuberty. Fluorescence *in situ* hybridization revealed 45,X[2]/46,X,der(Y).ish psu idic(Y)(q11.2)(SRY++,DYZ3++)[38] karyotype. A literature review identified eight case reports of mixed gonadal dysgenesis associated with 45,X/46,X,idic(Y)(q11.2). Neither sex phenotype nor short stature correlated with the 46,X,idic(Y)(q11.2) mosaic ratio.

## 1 Introduction

Isodicentric Y chromosomes [idic(Y)] are the most common structural abnormalities of the Y chromosome. The instability of idic(Y) during cell division results in the mosaic 45,X/46,X,idic(Y) karyotype (1). Depending on the different distribution of the 45,X cell line, phenotype varies and may include male infertility, Turner syndrome in females, ambiguous genitalia, gonadal dysgenesis, and short stature (2, 3). Here, we report a case of mixed gonadal dysgenesis in an 8-year-old boy with ambiguous genitalia, ectopic urethral opening, and short stature. Initial chromosomal analysis revealed 46,X,+mar karyotype. Subsequent investigation using fluorescence *in situ* hybridization demonstrated 45,X[2]/46,X,der(Y).ish psu idic(Y)(q11.2)(SRY++,DYZ3++)[38] karyotype.

## 2 Case report

**Figure 1:**
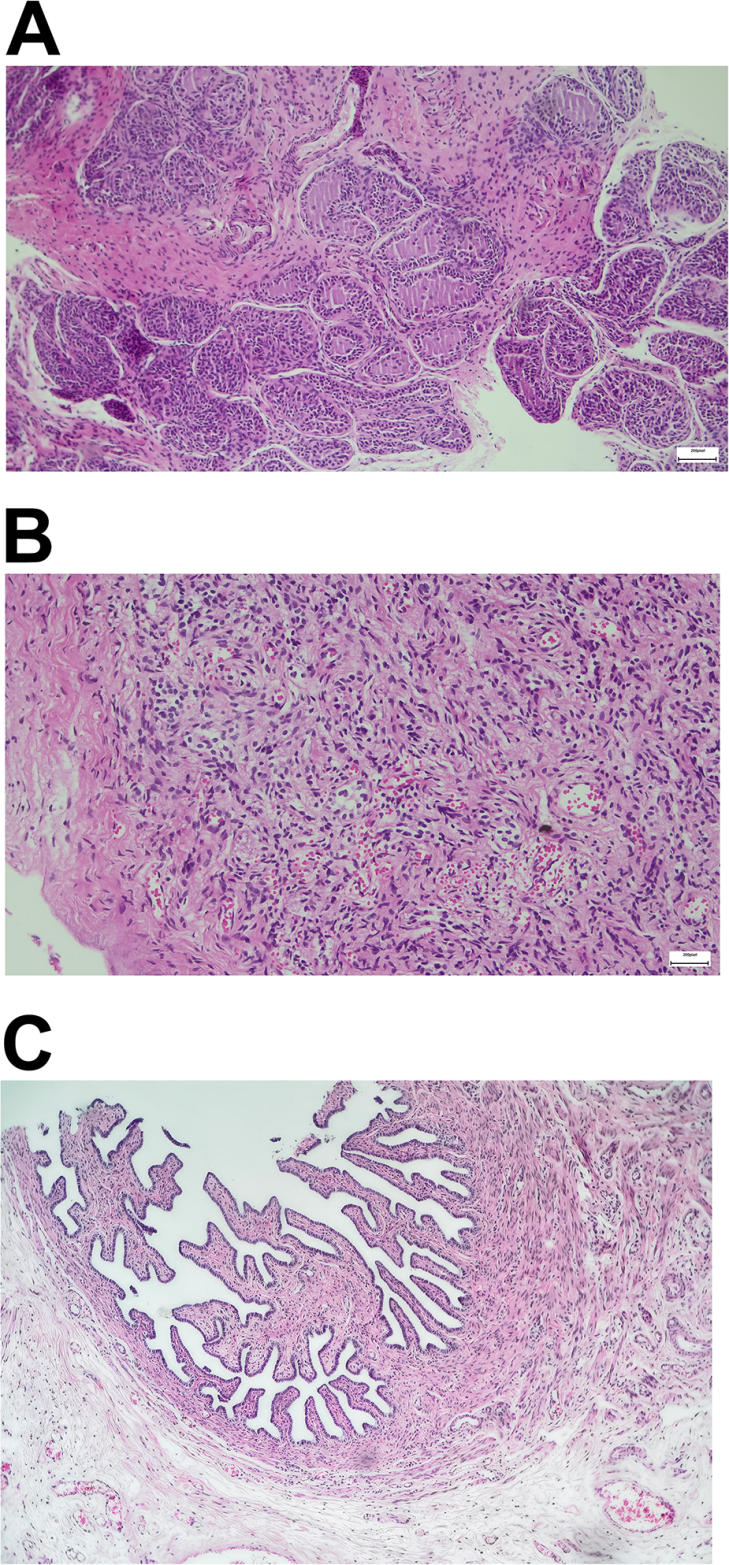
Pathologic examination after laparoscopic exploration. **(A)** Gonadal dysplasia of the right testis. **(B,C)** Ovarian stroma and right fallopian tube.

An 8-year-old boy presented with short stature (111.4 cm, −3.54 standard deviation; 19.5 kg, −2.11 standard deviation) for possible treatment with recombinant growth hormone. Upon inquiry, the parents disclosed *in vitro* fertilization birth and a diagnosis of mixed gonadal dysgenesis with a karyotype (46,X,+mar) at 14 months of age and a series of corrective surgeries. Both parents had normal karyotypes. Past medical records documented were as follows: (1) hypospadias and bilateral cryptorchidism; (2) dysplastic immature uterus; (3) surgery to correct hypospadias; (4) high ligation of processus vaginalis; and (5) removal of dysplastic testis on the right side (containing ovarian tissue upon pathological examination; Figure 1) and orchiopexy on the left side.

Physical examination is consistent with surgical history and was unremarkable otherwise except for the short stature. Upon ultrasound examination, the remaining testis appeared normal (17 × 11 × 7 mm, +0.17 standard deviation). The results of the laboratory tests were consistent with the male phenotype at prepuberty and excluded idiopathic growth hormone deficiency (Table 1).

**Table 1:** Key laboratory test results.

	Serum concentration	Normal reference range
Anti-Müllerian hormone	>18.00 ng/ml	2.04–19.22 ng/ml
Inhibin B	40.84 pg/ml	21.00–166.00 pg/ml
Luteinizing hormone	<0.1 IU/L	<0.1 IU/L
Follicle-stimulating hormone	1.34 IU/L	1.5–12.4 IU/L
Estradiol	<18.35 pmol/L	<18.35 pmol/L
Progesterone	<0.159 nmol/L	<0.474 nmol/L
Testosterone	<0.087 nmol/L	<0.087 nmol/L
Dihydrotestosterone	<0.16 nmol/L	0.55–2.72 nmol/L
Dehydroepiandrosterone	0.59 ng/ml	0.3–2.5 ng/ml
Androstenedione	0.45 nmol/L	<4.01 nmol/L
Peak growth hormone	23.5 ng/ml	>10 ng/ml

AMH was tested using a chemiluminescence method. Growth hormone stimulation test was conducted with arginine and analyzed using a chemiluminescence method.

**Figure 2:**
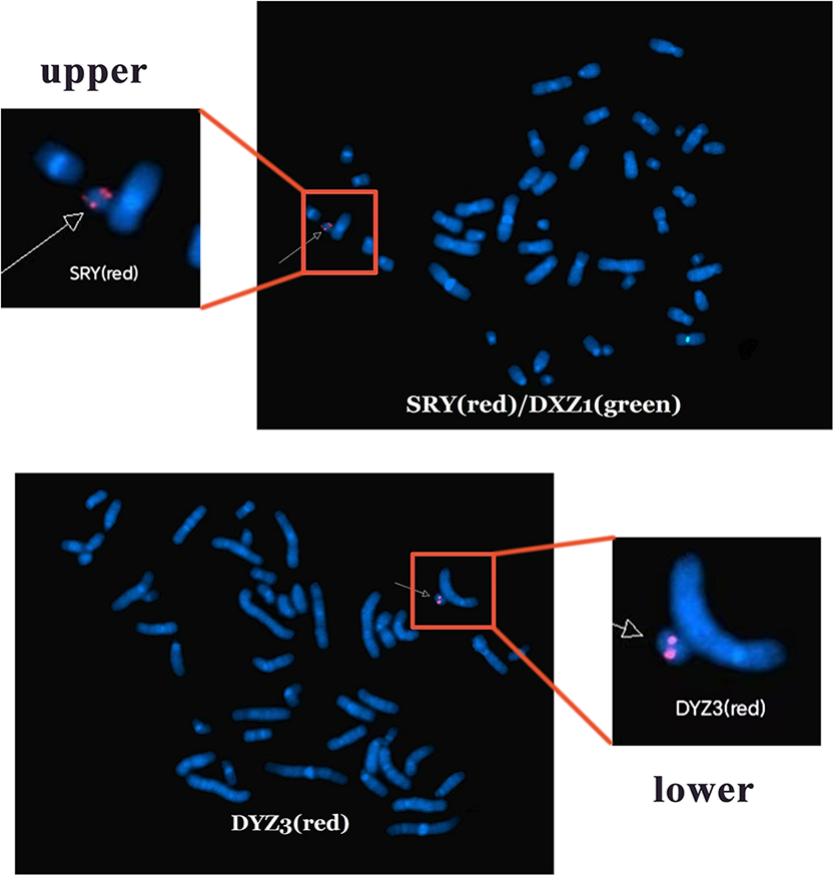
Fluorescence *in situ* hybridization analysis. SRY shows two red signals, DXZ1 shows one green signal (upper), and DYZ3 shows two red signals (lower).

**Figure 3:**
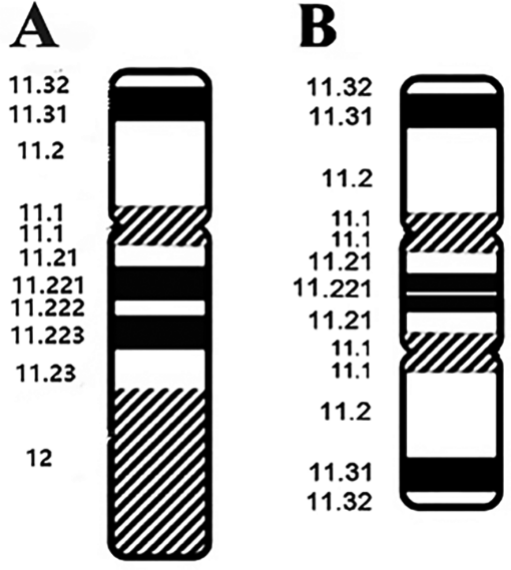
A schematic diagram of the isodicentric Y chromosome. **(A)** Normal Y chromosome. **(B)** Dicentric idic(Y) (q11.2) Y chromosome. White lines: breakpoints.

Based on these findings, three probes (*SRY*, *DXZ1*, and *DYZ3*) were designed for FISH, which in turn revealed a 45,X[2]/46,X,der(Y).ish psu dic(Y)(q11.2)(SRY++,DYZ3++)[38] karyotype (Figure 2). A schematic diagram of the isodicentric Y chromosome is shown in Figure 3. Considering the normal level of growth hormone and the risk of malignant transformation associated with dysplastic immature uterus, we decided not to initiate treatment with recombinant growth hormone as the parents initially requested. The patient was subsequently lost to follow-up.

## 3 Discussion and conclusions

The Y chromosome contains key genes that are critical for male sexual development, including the sex-determining gene *SRY* and azoospermia factor (4). A deficient *SRY* gene has been associated with dicentric Y chromosomes (5). Despite the presence of two *SRY* and two SHOX genes, the index patient had mixed gonadal dysplasia and short stature, respectively (6). Phenotype variation across the patients is caused by different breakpoints and distribution of different tissues of 45,X cell line, especially the gonads. Furthermore, the timing of the mitotic loss of idic(Y) during gonadal ontogenesis and the proportion of *SRY*-positive pre-Sertoli cells in the gonad is probably more relevant than the postnatal proportion of the different mosaic clones (7). The haplotype duplication involving *SHOX* in the Yp11.32q11.221 region in the index patient is consistent with a previous study that described isolated *SHOX* microreplicates with short stature (8). However, the 45,X mosaic karyotype in the index patient could affect linear bone growth due to partial *SHOX* gene haplo-insufficiency.

We conducted a literature review of mixed gonadal dysgenesis associated with 45,X/46,X,idic(Y)(q11.2). A search in PubMed identified nine reported cases (9–17). All reported cases were characterized by gonadal dysgenesis, but sexual phenotype varied across the cases: female in five cases, male in three cases, and undetermined in one case (Table 2). Most notably, neither sex phenotype nor short stature correlated with the 46,X,idic(Y)(q11.2) mosaic ratio.

**Table 2:** Mosaic idic(Y)(q11.2) karyotypes in the literature.

Case	Sex	Age (years)	Phenotype	Karyotype in blood cells	46,X,psu idic(Y)(q11.2) ratio
Han et al. (14)	Female	16	Short stature (147 cm, −2.3SD), primary amenorrhea, bilateral breast dysplasias, bilateral streak ovaries	45,X[3]/46,X,psu idic(Y)(q11.2)[37]	92.5%
Jagannath et al. (16)	Female	42	Short stature (145 cm, −2.52 SD), shield chest, primary amenorrhea, hypertension, hyperlipidemia	45,X[25]/46,X,psu idic(Y)(q11.2)[5]	16.7%
Gole et al. (9)	Female	2.7	Short stature (81 cm, −3.32 SD), clitoromegaly	45,X/46,X,idic(Y)(q11.2)	90%
Smith et al. (11)	Female	66	Clitoromegaly, no breast development	45,X/46,X,dic(Y)(q11.2)	30%
Shimoda et al. (12)	Female	29	Ambiguous genitalia with clitoromegaly	45,X[13]/46,X,idic(Y)(q11.2)[17]	56.7%
Kawabe et al. (15)	Male	15	Short stature (145 cm, −3.71 SD), scrotal hypospadias	45,X[14]/46,X,psu idic(Y)(q11.2)[16]	53.3%
Yoshida et al. (10)	Male	28	Short stature (156 cm, −2.74 SD), azoospermia	45,X[7]/46,X,psu idic(Y)(q11.2)[33]	82.5%
Reddy et al. (13)	Undetermined	Infant	Mixed gonadal dysgenesis	45, X /46, X, psu idic (Y) (q11.2)	8%
Mekkawy et al. (17)	Male	Infant	Ambiguous genitalia, short stature (−3.3 SD), left streak ovary	46,X,idic(Y)(q11.2)[74]/45,X [24]/47,X, idic(Y)(q11.2)x2 [2]. ish idic(Y)(q11.2)	76%
Index patient	Male	8	Short stature (111. 4 cm, −3.54 SD), mixed gonadal dysgenesis	45,X [2]/46,X,psu idic(Y)(q11.2)[38]	95%

In summary, the mixed gonadal dysgenesis in the index patient was caused by *de novo* 45,X[2]/46,X,der(Y).ish psu idic(Y)(q11.2)(SRY++,DYZ3++)[38] karyotype. This case represents a valuable addition to the limited collection of Yq11.2 breaks in the literature.

## Data Availability

The raw data supporting the conclusions of this article will be made available by the authors, without undue reservation.
